# Effects of Simulated Nitrogen Deposition on Soil Net Nitrogen Mineralization in the Meadow Steppe of Inner Mongolia, China

**DOI:** 10.1371/journal.pone.0134039

**Published:** 2015-07-28

**Authors:** Xing-ren Liu, Jian-qiang Ren, Sheng-gong Li, Qing-wen Zhang

**Affiliations:** 1 Agricultural Clear Watershed Group, Institute of Environment and Sustainable Development in Agriculture, Chinese Academy of Agricultural Sciences, Beijing 100081, China; 2 Key Laboratory of Agricultural Environment, Ministry of Agriculture, P. R. China; 3 Institute of Agricultural Resources and Regional Planning, Chinese Academy of Agricultural Sciences, Beijing 100081, China; 4 Key Laboratory of Ecosystem Network Observation and Modeling, Institute of Geographic Sciences and Natural Resources Research, Chinese Academy of Sciences, Beijing 100101, China; Tennessee State University, UNITED STATES

## Abstract

Effects of simulated nitrogen (N) deposition on soil net nitrogen mineralization (NNM) were examined *in situ* during two growing seasons, using the resin-core technique in the semiarid meadow steppe in Inner Mongolia, China. The aim of this study is to clarify the effect of N levels (0, 10, and 20 kg N ha^−1^yr^−1^) and forms (NH_4_^+^ and NO_3_^-^) on soil mineral N and NNM. Our results showed that N levels had no significant differences on soil mineral N and NNM. In the first year, three N treatments ((NH_4_)_2_SO_4_, NH_4_Cl and KNO_3_) increased soil NH_4_^+^ concentrations but had no significant effects on soil NO_3_^-^ concentrations. In the second year, (NH_4_)_2_SO_4_ treatment increased soil NO_3_^-^ concentrations, NH_4_Cl and KNO_3_ treatments decreased them. Three N treatments significantly decreased soil NH_4_^+^ concentrations in the later stages of the second year. As for the soil NNM, three N treatments had no significant effects on the rates of soil NNM (*R*_m_) and net nitrification (*R*_n_) in the first year, but significantly decreased them in the second year. The contribution of N addition to *R_m_* was higher from (NH_4_)_2_SO_4_ than from NH_4_Cl and KNO_3_. However, Soil *R*_m_ was mainly affected by soil water content (SWC), accumulated temperature (T_a_), and soil total N (TN). These results suggest that the short-term atmospheric N deposition may inhibit soil NNM in the meadow steppe of Inner Mongolia.

## Introduction

The global nitrogen (N) deposition has increased from 34 to 105 Tg yr^−1^ since before 1860 and is expected to double in the next 25 years because of the anticipated increase in human activities [1–4]. In China, the average annual dry and wet N deposition is estimated at 12.9 kg N ha^−1^ yr^−1^, and it has been increasing at a rate of 0.17 kg N ha^-1^ yr^−1^ or 1.47% per year [5]. Elevated atmosphere N deposition has been considered as an important factor influencing the N cycle processes especially on mineralization, nitrification and denitrification in terrestrial ecosystems [2,3,6]. Therefore, it is important to better understand the impacts of N deposition on N transformation in N-limited natural ecosystem, and to accurately evaluate the changes in ecosystem productivity and capability to sequester N induced by elevated N deposition.

Soil organic N mineralization, as one of the major processes of N cycle plays an important role in determining soil N availability and net primary productivity [7,8]. Net nitrogen mineralization (NNM) is the outcome of two concurrent and opposite processes: gross N mineralization and gross N immobilization turnover. The actual availability of inorganic N depends on the rate of NNM and its transport through the soil [9,10].

Simulated atmospheric N deposition experiments on soil NNM have been conducted in some forest ecosystems. Several prior studies have discussed whether N additions have a positive [11–16] or a negative effect on NNM [17–19]. Some of them were measured by laboratory incubation, which did not agree well with the results from the field observation. Most of N inputs were mixed type of N fertilizer, NH_4_NO_3_, which will not help understanding of the single effects of different N forms on NNM. The response of soil NNM to N deposition depends largely on the input of N forms and concentrations, the balance between production processes of H^+^ (nitrification, plant uptake, and the fixation of NH_4_^+^) and its consumption (denitrification, plant uptake, and the fixation of NO_3_^-^), and the buffering capacity of soils [6,8,20]. However, the effects of N deposition on NNM remain far from certain especially in China’s temperate grassland which comprises nearly 12.5% of the global grassland areas.

To improve our understanding of the responses of NNM to the elevated atmosphere N deposition in China’s temperate grassland, we conducted an *in situ* N fertilization addition experiments in the semiarid meadow steppe in Inner Mongolia, China in May 2008. The experiment lasted for two years, and it involved different forms and levels of N. The objective of these experiments was to explore the effects of the amounts and forms of N addition on soil inorganic N and rates of NNM *in situ* in the meadow steppe of Inner Mongolia, China.

## Materials and Methods

### Site description

**Fig 1:**
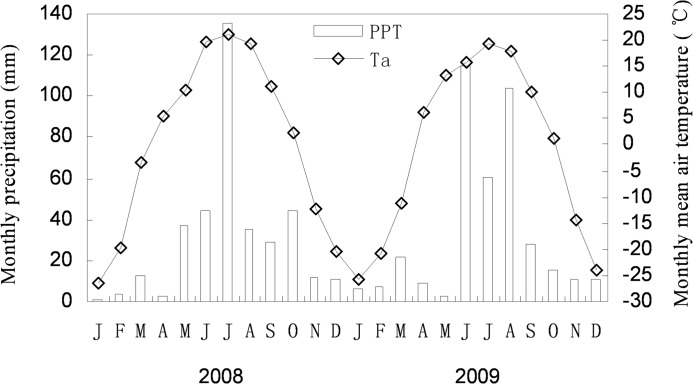
Air temperature and precipitation at the meteorological station close to the study area in 2008 and 2009.

The simulated N deposition experiment was conducted in one of the grazing-excluded experimental plots of the meadow steppe at the Inner Mongolia Grassland Ecosystem Research Station of the Chinese Academy of Agricultural Sciences. The site is located in Hulun Buir City, Inner Mongolia, China, 49°19′–49°20′N latitude, 119°55′–119°58′E longitude, 628–649 m above the sea level. The average annual dry and wet N deposition is estimated at 8.5 kg N ha^−1^ yr^−1^. The selected plot was fenced in 1997. The mean annual precipitation at the site is 350–400 mm. Precipitation occurs mainly from June to September. Mean annual temperature ranges from –5 to –1°C. The ground is covered by snow in most of the winter season from November to April of the following year. Annual cumulative temperature of ≥10°C is 1680–1800°C, and the frost-free period is around 100 days. Air temperature and precipitation in 2008 and 2009 are shown in Fig 1.

The soil type at the site is classified as chestnut according to the Chinese classification, or Haplic Arenosol according to the FAO classification. Total N, inorganic N, C/N ratios, pH values and soil bulk density are shown in Table 1. One of the main plant community types (the climax community) in the area is the *Stipa baicalensis*. The grass community is mainly composed of *S*. *baicalensis*, *Leymus chinensis*, *Carex pediformis*, *Artemisia tanacetifolia*, *Melissitus ruthenica*, *Bupleurum scorzonerifolium*, *Vicia amoena*. Among them, *S*. *baicalensis* and *L*. *chinensis* are the dominant species.

**Table 1:** Soil physiochemical properties of 0–20 cm soil in the sampling site.

Total Organic C(%)	DOC(mg kg^-1^)	Total N(%)	NH_4_^+^-N(mg kg^-1^)	NO_3_^—^N(mg kg^-1^)	C/N	pH	Soil bulk density(g cm^-3^)
5.1	122.9	0.20	13.4	4.6	25.5	5.9	1.22

### Experimental design

In May 2008, a plot was selected for N deposition experiment in the fenced meadow steppe. Three N fertilizers ((NH_4_)_2_SO_4_, NH_4_Cl, and KNO_3_) were used as three levels of control (CK, 0 kg N ha^−1^ yr^−1^), low N (LN, 10 kg N ha^−1^ yr^−1^), and high N (HN, 20 kg N ha^−1^ yr^−1^). The fertilized amount was set to simulate a future increase in the atmospheric N deposition by 1- and 2- fold. Each N treatment was replicated three times, a total of 21 sub-plots of 3 m × 4 m in size were set up in a randomized block design. The adjacent sub-plots were separated by a 2 m wide buffer strip to minimize the disturbance from neighboring treatments. One year’s amount of added N was divided into two equal portions: one portion in the growing season (from May to October) and the other portion in the non-growing season. Over the growing season, N fertilizers were added on around 10th of every month as 6 equal doses. Duo to the severe weather conditions in the non-growing season, N fertilizers were all applied in one time in November. N fertilizer was dissolved in 1 L deionized water, and sprayed on each sub-plot, and the control plots received 1 L deionized water only. So 7 L additional water was added to each sub-plot per year. No significant differences in general soil properties were observed between the treatments before N application.

### *In situ* net N mineralization

The experiment was carried out from May 2008 to October 2009 (2 integral growing seasons) using the resin-core technique [21,22], which was similar to that described by Liu et al. [23]. The method confined soil cores *in situ* in an open tube, with an anion exchange resin bag at the bottom to intercept any leached N. At each sampling, four duplicate samples of two paired samples of soil were taken in each subplot. The PVC tubes were sharpened in the outside at the bottom to avoid compression of grassland soil. After the aboveground vegetation was removed, one of a pair of PVC tubes (5 cm in diameter and 17 cm long) was inserted 15 cm into the soil. The soil was taken to the laboratory for the determination of the extractable N. The other tube was inserted 15 cm into the soil to confine a soil core, and then 2 cm soil layer was excavated from the bottom of the tube. The excavated soil was put into a filter paper, one resin bag, filter paper, and one cylindrical block of gypsum in order (the height of resin bag and plaster is about 2 cm). The resin bag was made of nylon and containing 10 g of anion exchange resin beads. The tube was then returned to its original position, and left for *in situ* incubation for about 4 weeks. When it was removed and further tubes were driven into the soil to repeat the cycle until the experiment ended on 15 October. All the initial and incubated soil samples and the collected resin bags were stored in refrigerator at 4°C temporarily, and extracted within 24 h.

### Soil samples and resin bags extraction and chemical analysis

After stones had been removed by hand, all soil samples were thoroughly mixed, homogenized, and sieved through a 2 mm sieve. Mineral N was extracted with 0.01 M CaCl_2_ solution. The resin bags were extracted with 1 M NaCl solution (resin: NaCl 1: 5 w/v). The filtrates of soil and the solution of extracted resin bags were kept frozen before they were analyzed on an automated flow injection analysis (Braun & Lübbe, Norderstedt, Germany). Rates of net ammonification (*R*_a_), net nitrification (*R*_n_), and net mineralization (*R*_m_) were calculated from measured changes in inorganic N content divided by the time interval [23]. Soil water content (SWC) was determined gravimetrically by oven-drying at 105°C for 24 h. Soil temperature at 10 cm depth was measured with Model SN 2202 digital thermo detector at the time of sampling.

Total soil organic carbon (TOC) and TN were analyzed using a Multi N/C 2100 analyzer (Analytik Jena, Germany). Soil dissolved carbon (DOC) was assayed following the procedures presented by Ghani et al. (2003) [24]. Soil pH values were determined in water (water: soil = 2.5: 1) suspension. Soil bulk density was measured using the core method.

The peak aboveground and root biomasses were measured in mid-August in both years. Aboveground biomass was measured by harvesting vegetation over a 25 cm × 25 cm area (n = 5). Root biomass was measured by a 10 cm diameter soil cores collected to 40 cm depth, because more than 95% of the root biomasses were in this depth. At each sampling, 10 cores were collected on each plot. Live roots, carefully removed from the soil cores, were rinsed with water. All plant materials were dried in an oven at 60°C to constant weight.

### Statistical analysis

Statistical analyses were conducted using the SPSS 16.0 package. Means (*n* = 3) and standard errors (SE) were calculated. We used three way repeated measures multivariate analysis of variance (MANOVA) with N forms, N levels and sampling time as main effects to test differences in NH_4_^+^-N, NO_3_^–^-N, *R*_m_, *R*_n_, and *R*_*a*_. Relationships between *R*_m_ and soil parameters were tested using Pearson correlation analysis. Statistical significant differences were set at P values <0.05 unless otherwise stated.

## Results

### Seasonal dynamics of mineral N concentration

**Fig 2:**
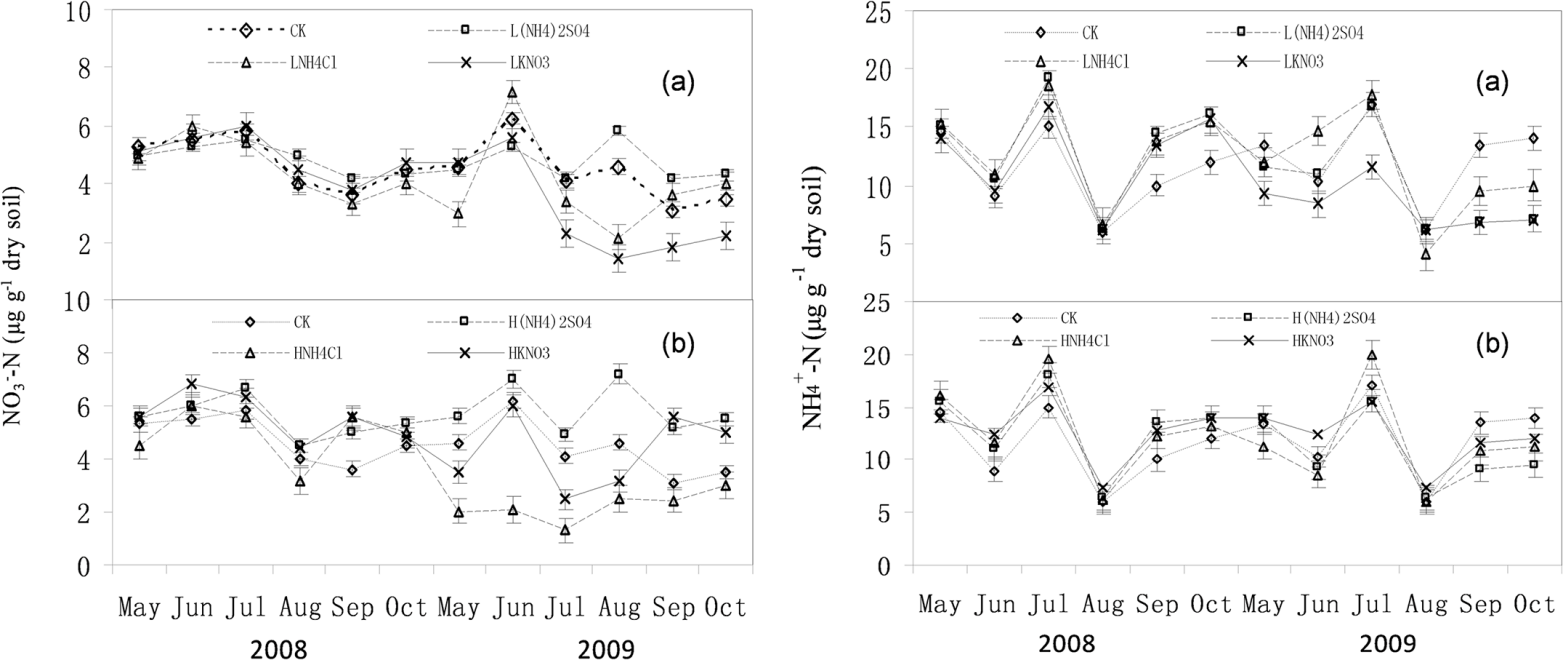
The seasonal variations of NO_3_^-^-N and NH4+-N in LN (a) and HN treatments (b) during the growing seasons of 2008 and 2009.

The seasonal patterns of NO_3_^—^N were similar between LN (a) and HN (b) treatments with one peak on 15 July 2008 and two peaks on 15 June and 12 August 2009 (Fig 2). Levels rather than forms of N addition had subtle effects on soil NO_3_^—^N (Table 2, P>0.05) except for NH_4_Cl (P<0.05) in 2009. In 2008, there were no significant differences between N addition treatments and the control. In 2009, H(NH_4_)_2_SO_4_ treatment increased NO_3_^—^N concentrations compared to CK, while HNH_4_Cl and HKNO_3_ treatments decreased them significantly (P<0.05). The reduction effect of HNH_4_Cl on NO_3_^—^N concentrations was more obvious (P<0.05).

**Table 2:** Results of F tests based on repeated measures ANOVA on effects of N level, N forms, and their interactions on soil mineral N, *R*_*m*_, *R*_*n*_, and *R*_*a*._

Variations	Growing season of 2008					Growing season of 2009				
	NH_4_^+^-N	NO_3_^—^N	*R*_*m*_	*R*_*n*_	*R*_*a*_	NH_4_^+^-N	NO_3_^—^N	*R*_*m*_	*R*_*n*_	*R*_*a*_
Time (T)N level (L)	5.75*2.11	4.25*1.56	6.11*2.83	6.35*2.56	5.87*3.01	5.78*1.98	5.09*2.02	7.23*2.71	6.58*2.62	6.41*3.51
N form (F)	1.07	2.33	1.88	1.76	2.23	3.56	3.01	4.89*	5.07*	5.88*
T×L	1.10	0.96	1.23	1.07	0.99	1.26	1.55	0.89	1.42	0.76
T×F	2.05	1.67	2.00	1.54	1.76	1.87	2.13	1.17	1.53	0.75
L×F	1.05	1.34	1.32	1.85	1.11	1.52	2.26	0.99	1.06	1.63
T×L×F	1.18	0.93	0.87	0.56	0.32	1.76	1.64	0.52	0.39	0.47

*: statistically significant at P < 0.05

The seasonal patterns of NH_4_^+^-N were similar between LN (a) and HN (b) treatments, with two peaks occurred in July in both years. N levels had subtle effects on soil NH_4_^+^-N (Table 2, P>0.05), except for KNO_3_ treatments in 2009. LKNO_3_ treatment decreased NH_4_^+^-N concentrations significantly compared to CK (P<0.05), but HKNO_3_ treatment had no significant effects on them. In 2008, there were no significant differences among treatments, while in the later stages of 2009, three LN treatments decreased NH_4_^+^-N concentrations. The suppression effect of LKNO_3_ was the most obvious among the three LN treatments. In 2008, the three HN treatments tended to increase NH_4_^+^-N concentrations, but there were no significant differences between N addition treatments and control (P>0.05). In the later stages of 2009, the three HN treatments all decreased NH_4_^+^-N concentrations. Contrary to the LN treatments, ammonium N fertilizers addition had more reduction effects than nitrate N fertilizers on soil NH_4_^+^-N concentrations.

### Net nitrification rate

**Fig 3:**
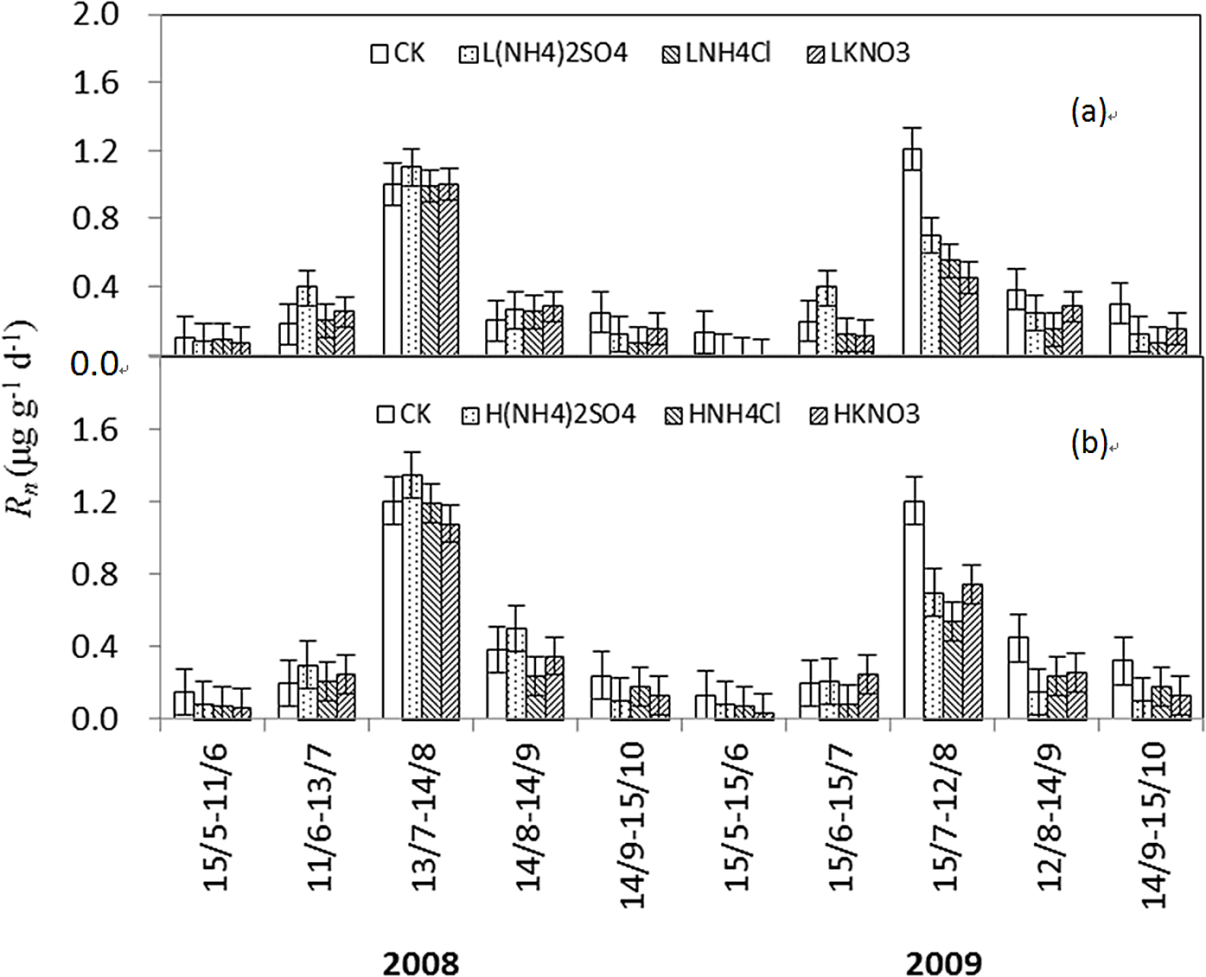
The seasonal variations of *R*_*n*_ in LN (a) and HN treatments (b) during the growing seasons of 2008 and 2009.

The seasonal patterns of daily *R*_n_ were similar between LN and HN treatments with two peaks in July in both years (Fig 3). In the LN treatments, *R*_n_ ranged from 0 to 1.21 μg g^–1^ d^–1^ and varied significantly during the two growing seasons. The incubation period had significant effects on *R*_n_ (P<0.05). In 2008, the three LN treatments had no significant effects on *R*_n_, while in the later stages of 2009, they decreased *R*_n_ significantly. L(NH_4_)_2_SO_4_, LNH_4_Cl, and LKNO_3_ treatments decreased *R*_n_ by 12.1–42.6%, 13.3–54.5%, and 13.0–62.8%, respectively, compared to CK. The reduction effect was most obvious in the warm and rainy July of 2009. However, there were no significant differences in *R*_n_ among each LN treatment.

In the HN treatments, *R*_n_ ranged from 0.03 to 1.35 μg g^–1^ d^–1^ and varied significantly during the two growing seasons (Fig 3). In 2008, H(NH_4_)_2_SO_4_ treatment tended to increase *R*_n_ during the incubation period from 11 June to 13 July, 13 July to 14 August, and 14 August to 14 September, but HNH_4_Cl and HKNO_3_ treatments had no significant effects on *R*_n_. In the later stages of 2009, the three HN treatments had significant reduction in *R*_n_. H(NH_4_)_2_SO_4_, HNH_4_Cl, and HKNO_3_ treatments decreased *R*_n_ by 14.1–66.2%, 13.2–55.4%, and 10.0–63.7% compared to CK, respectively. The reduction effects were most obvious in July of 2009. H(NH_4_)_2_SO_4_ treatment had a stronger inhibition on *R*_n_ than HNH_4_Cl and HKNO_3_ treatments.

### Net ammonification rate

**Fig 4:**
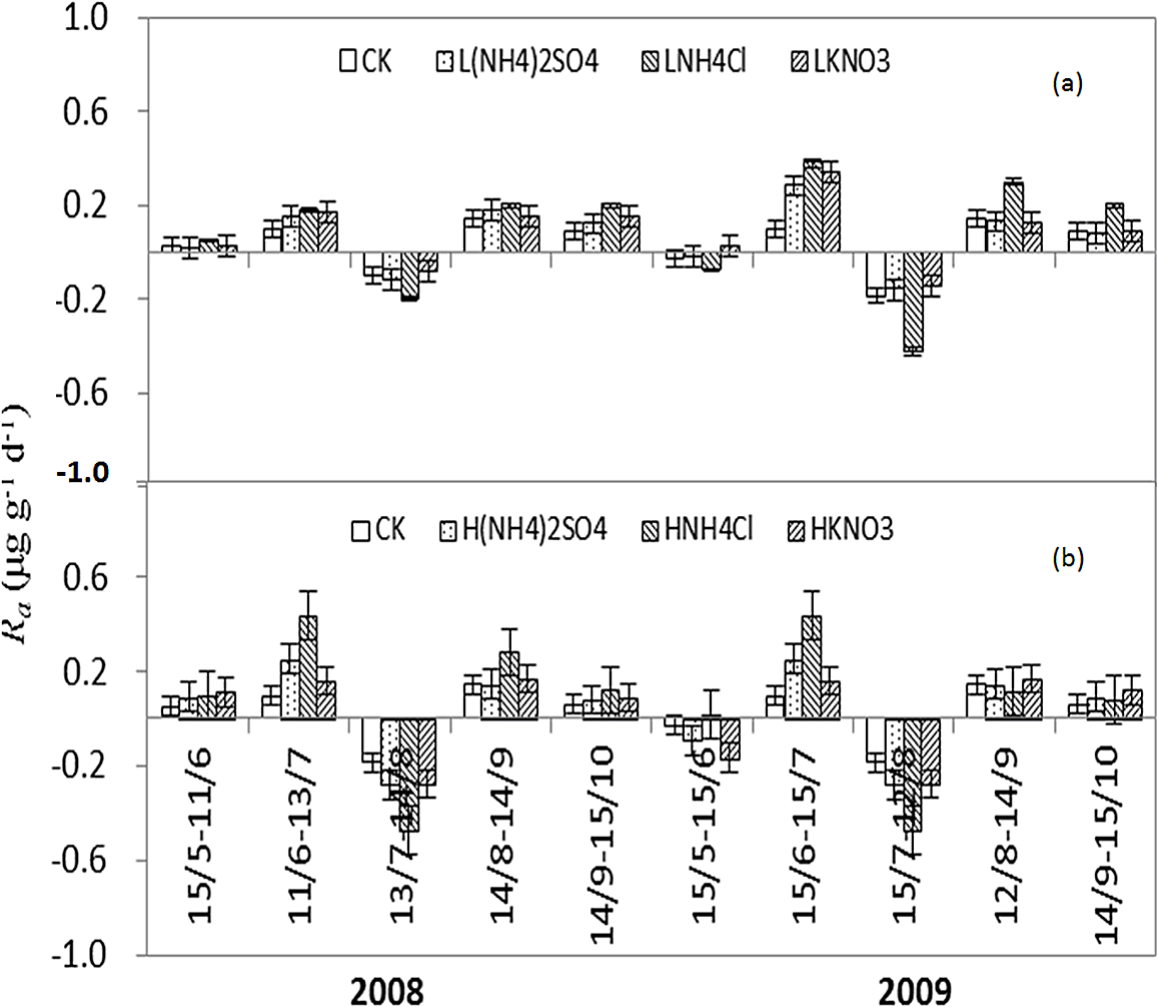
The seasonal variations of *R*_*a*_ in LN (a) and HN treatments (b) during the growing seasons of 2008 and 2009.

The seasonal patterns of daily *R*_*a*_ were similar between LN and HN treatments with three negative values in July 2008 and May and July 2009 (Fig 4). In the LN treatments, *R*_*a*_ ranged from -0.19 to 0.30 μg g^–1^ d^–1^ and varied significantly during the two growing seasons. The incubation period also had significant effects on *R*_*a*_ in all treatments (P<0.05). During the two growing seasons, the LNH_4_Cl treatment significantly increased *R*_*a*_, except for the negative values during the period from 13 July to 14 August 2008 and from 15 July to 12 August 2009. The promotion effects of LNH_4_Cl on *R*_*a*_ in 2009 were greater than that in 2008, and the effects were most obvious in June and August. LNH_4_Cl treatment increased *R*_*a*_ by 42.8–122.0% in 2008 and 114.0–288.0% in 2009. L(NH_4_)_2_SO_4_ and LKNO_3_ treatments had no significant effects on *R*_*a*_.

In the HN treatments, *R*_*a*_ ranged from -0.47 to 0.44 μg g^–1^ d^–1^ and varied significantly during the two growing seasons. The HNH_4_Cl treatment significantly increased *R*_*a*_, except for the negative values during the periods from 13 July to 14 August 2008 and from 15 July to 12 August 2009. Contrary to the LNH_4_Cl treatment, the promotion effect of HNH_4_Cl on *R*_*a*_ was greater in 2008 than in 2009. In the later period of 2009, there was no significant difference in *R*_*a*_ between treatments.

### Net N mineralization rate

**Fig 5:**
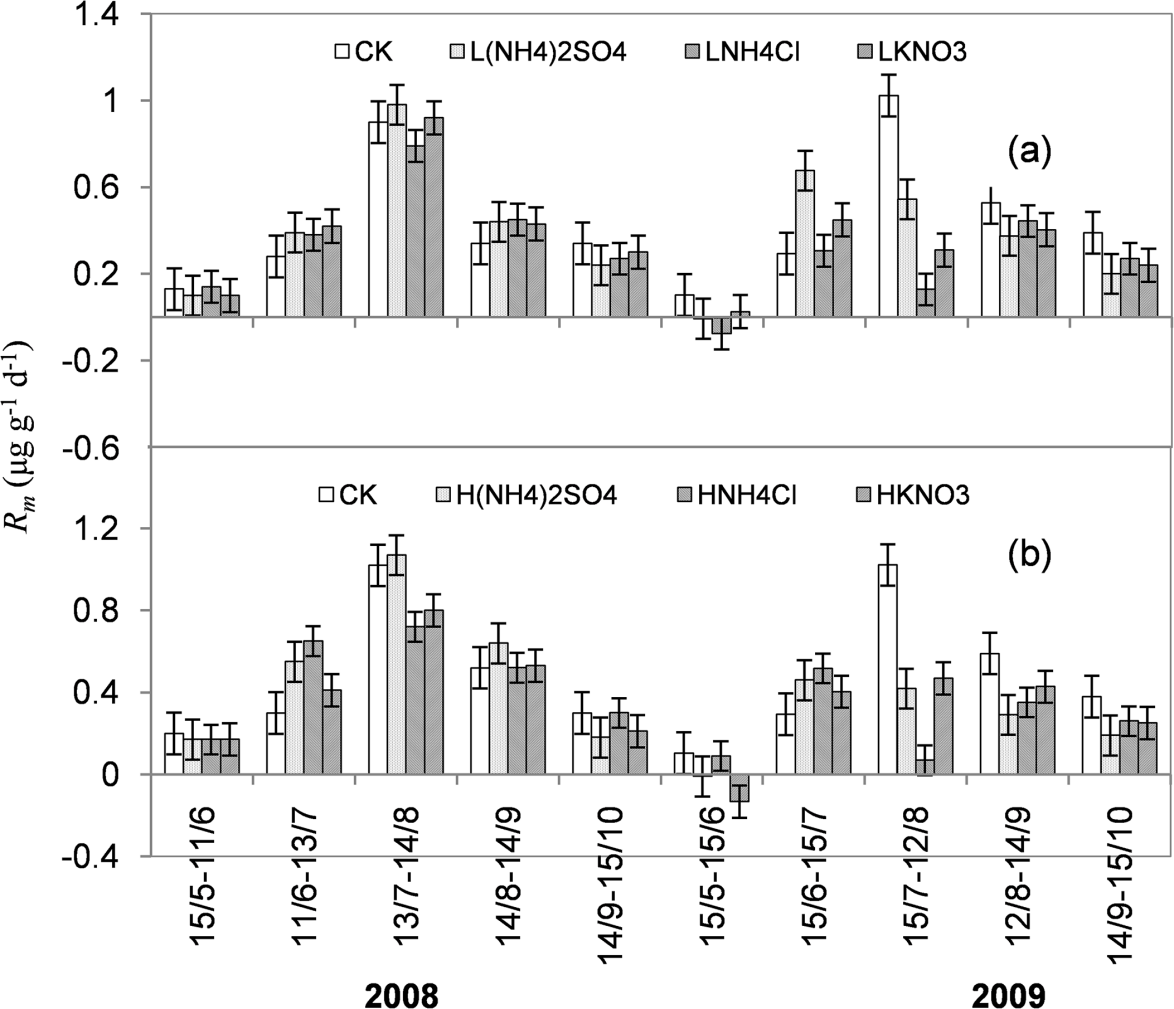
The seasonal variations of *R*_*m*_ in LN (a) and HN treatments (b) during the growing seasons of 2008 and 2009.

Given that *R*_n_ was far greater than *R*_*a*_ in values, the seasonal variations of *R*_m_ were similar to *R*_n_. The seasonal patterns of daily *R*_*m*_ were also similar between LN and HN treatments with two peaks in July of 2008 and 2009. In the LN treatments, *R*_*m*_ ranged from -0.08 to 1.02 μg g^–1^ d^–1^, and the incubation period had significant effects on *R*_*m*_ in all treatments (Fig 5, P<0.05). In 2008, the three N treatments had no significant effects on *R*_m_. In the later period of 2009, they significantly decreased it. L(NH_4_)_2_SO_4_, LNH_4_Cl, and LKNO_3_ treatments decreased *R*_m_ by 12.1–110.2%, 17.0–180.0%, and 24.5–70.3%, respectively, compared to CK. The most obvious reduction effects also occurred in July. However, there were no significant differences in *R*_m_ among the LN treatments except for the incubation period from 15 June to 15 July.

In the HN treatments, *R*_*m*_ ranged from -0.13 to 1.07 μg g^–1^ d^–1^, and varied significantly during the two growing seasons (Fig 5, P<0.05). In 2008, H(NH_4_)_2_SO_4_ treatment tended to increase *R*_m_ except for the beginning and the end of the growing season. HNH_4_Cl and HKNO_3_ treatments showed the same trend in *R*_m_, with increased *R*_m_ in June, and decreased *R*_m_ in July, at comparable magnitude to that in CK. In 2009, the three HN treatments significantly decreased *R*_*m*_ except for the period from 15 June to 15 July. H(NH_4_)_2_SO_4_ treatment had the most obvious reduction effect, especially in July. H(NH_4_)_2_SO_4_, HNH_4_Cl, and HKNO_3_ treatments reduced *R*_m_ by 36.7–58.8%, 13.0–93.1%, and 16.7–53.9%, respectively, compared to CK. However, there were no significant differences between HNH_4_Cl and HKNO_3_ treatments.

### Aboveground biomass and root biomass

**Fig 6:**
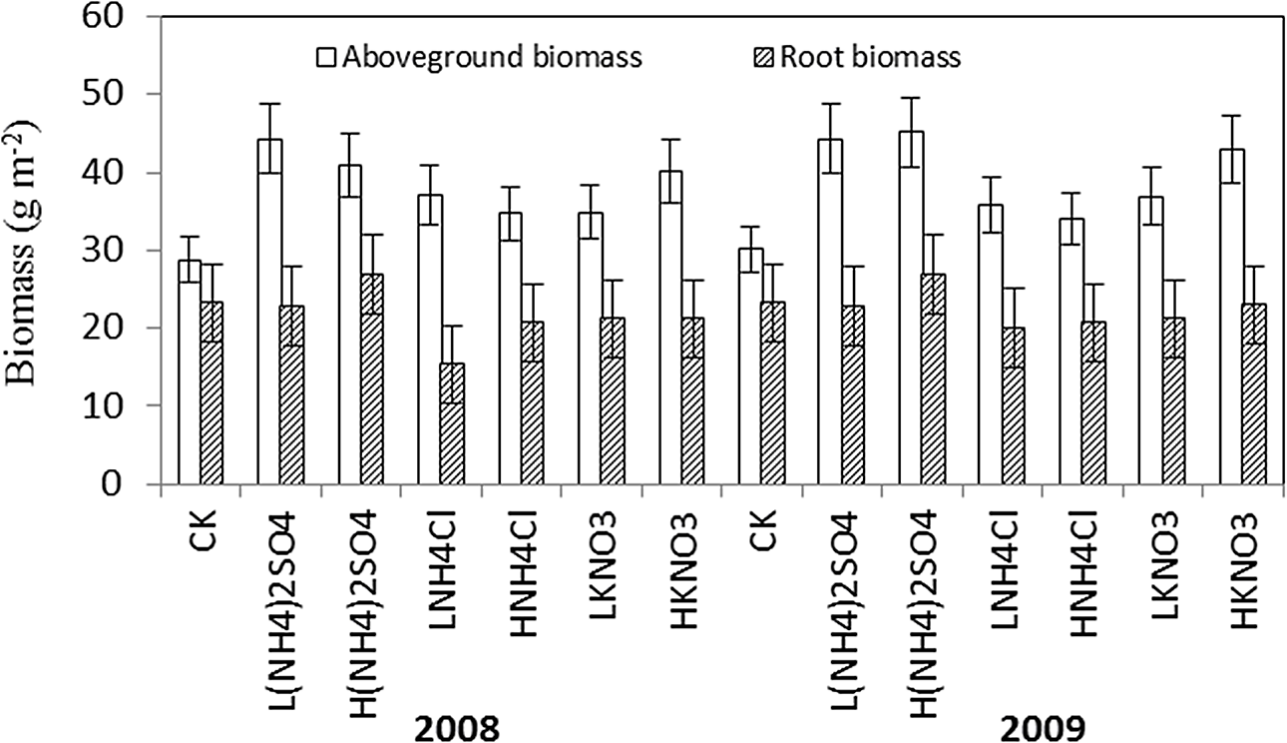
The aboveground biomass and root biomass of different treatments in August of 2008 and 2009.

In either the LN or HN treatments, N addition caused significant increases in aboveground biomasses (Fig 6, P<0.05), but not root biomasses. In 2008, L(NH_4_)_2_SO_4_, H(NH_4_)_2_SO_4_, LNH_4_Cl, HNH_4_Cl, LKNO_3_, and HKNO_3_ treatments increased aboveground biomasses by 53.8%, 41.7%, 28.8%, 20.5%, 21.2%, and 39.6%, respectively. In 2009, they increased aboveground biomasses by 47.2%, 49.8%, 18.9%, 13.0%, 22.6%, and 42.9%, respectively. However, there were no significant differences between N levels (P>0.05). The increases in aboveground biomasses of (NH_4_)_2_SO_4_ treatments were greater than that of NH_4_Cl and KNO_3_ treatments, but there were no significant differences between LN and HN treatments.

### Relationships between *R*_*m*_ and environmental factors

Our results showed that soil *R*_*m*_ was significantly positively correlated with soil SWC and the effective accumulated temperature (T_a_) in each treatment (Table 3). The variations of soil SWC and T_a_ could explain more than 58.1% and 80.5% of the variation in *R*_*m*_, respectively (Table 3, P<0.05). Also, soil *R*_*m*_ was positively correlated with the soil TN rather than TOC. The variation of TN could explain more than 53.4% of the variation in *R*_*m*_. Soil *R*_*m*_ was negatively but not significantly correlated with C/N of soil organic matter (SOM) (Table 3, P>0.05). There were positive but insignificant correlations between *R*_*m*_ and soil pH values (Table 3, P>0.05).

**Table 3:** Pearson’s linear correlation coefficient (r) between environmental factors and *R*_*m*_ under different treatments.

Item	CK	H(NH_4_)_2_SO_4_	L(NH_4_)_2_SO_4_	HNH_4_Cl	LNH_4_Cl	HKNO_3_	LKNO_3_
SWC (%)T_S_ (°C)T_a_ (°C)TN (%)TOC (%)Soil C:N	0.628*0.3410.791*0.615*0.485–0.490	0.661*0.4530.818*0.634*0.543–0.432	0.654*0.4320.821*0.650*0.511–0.516	0.595*0.5210.805*0.534**0.327–0.509	0.581*0.4110.887**0.651**0.153–0.498	0.621*0.4540.876**0.647**0.138–0.671	0.609*0.4910.855**0.621*0.012–0.457
Soil pH	0.381	0.468	0.381	0.474	0.597*	0.405	0.313

T_S_: soil temperature; T_a_: accumulated temperature

*, **: statistically significant at P<0.05, 0.01.

## Discussion

### The effects of N addition on mineral N

Our results showed that in both the HN and LN treatments, the additions of (NH_4_)_2_SO_4_ increased soil NO_3_^-^ concentrations, NH_4_Cl and KNO_3_ decreased soil NO_3_^-^ concentrations in the second year. These results are very similar to the results from the long-term fertilization experiment in the neutral purple paddy soil ecosystem reported by Qin et al. [25], and also similar to the results of Zhang et al. [26] from experiments with three different soil types in grassland. Besides the specific NH_4_^+^ or NO_3_^-^ effects, the salt effects of SO_4_^2-^ and Cl^-^ may be a reasonable explanation for the increase or decrease in NO_3_^-^ concentrations. We found SO_4_^2-^ promoted nitrification, and Cl^-^ had significant inhibitory effects on nitrification. Application of different NH_4_^+^-N fertilizer, soil chemical and physical properties would change because of salt effects (such as osmotic pressure, pH value). (NH_4_)_2_SO_4_ in soil solution is easier to produce more H^+^ than NH_4_Cl, but the osmotic pressure of NH_4_Cl in soil solution is greater than that of (NH_4_)_2_SO_4_ [27]. Many researches have indicated that NH_4_Cl has direct inhibitory effects on nitrification [27,28]. Few researches, however, has not examined the inhibition mechanism. Therefore, the mechanism of the inhibitory effects of NH_4_Cl on nitrification needs to be further studied.

Our study also showed that the promotion of (NH_4_)_2_SO_4_ fertilizer to soil NO_3_^—^N concentration was higher than KNO_3_ fertilizer, and the inhibition of KNO_3_ fertilizer on soil NH_4_^+^-N concentration was higher than (NH_4_)_2_SO_4_ fertilizer (Fig 2). Generally, NO_3_^—^N and NH_4_^+^-N fertilizers should have opposite effects on soil mineral N because of their opposite ion charges [29]. The less sensitivity of soil NO_3_^—^N concentration to KNO_3_ amendment is because that NO_3_^-^ are very mobile in soil owing to their negative charge[30]. Moreover, NO_3_^-^ may not be present in the treated plots as long as NH_4_^+^, which are positively charged and are more strongly adsorbed onto exchange sites in the soil matrix [29]. Also, the K^+^ can exchange NH_4_^+^ from the exchange sites in the soil and then release NH_4_^+^ [27]. This explanation is supported by our results of the accumulation of soil NH_4_^+^-N and NO_3_^—^N with N addition (Fig 2).

In this study, we did not observe significant effects of N addition on inorganic N concentrations in the first year, and N levels on inorganic N concentrations in the second year. Generally, the dynamics of soil NO_3_^—^N and NH_4_^+^-N concentrations under N additions are mainly determined by the inorganic N input (N addition and mineralization) and losses to plant uptake. The addition of N fertilizers significantly increased aboveground biomasses in our experiment (Fig 6, P<0.05), previous study also reached the same conclusion [31]. These results suggested that the loss of soil inorganic N through the uptake of vegetation offsets the input N and soil organic N mineralization. On the other hand, the meadow steppe soil is N-limited and has a strong capacity to immobilize exogenous N, perhaps the N level in our experiment is not enough to affect the mineral N concentration in the soil. We attributed the no responses to the short-term phenomenon. In a short term, the abundance and activity of soil microorganisms are regulated more by plant species than by direct effect of N input [31,32]. The effects of N input on soil N pool are decided by two processes: 1) the increased nitrification would increase the activity of soil N, accelerate the NO_3_^-^ loss, and reduce the soil N pool [19]. 2) The increased available N would inhibit production of lignin degrading enzyme, allowing NO_3_^—^N and NH_4_^+^-N to combine with lignin and phenolic compounds to form stable compounds not easy for decomposition. This process would reduce N release rate, and thereby improve soil N accumulation [20,33]. The soil N accumulation varies with N deposition according to the balance of the processes 1 and 2.

### The effects of N addition on *R*_m_, *R*_n_, and *R*_a_

In the later period of 2009, (NH_4_)_2_SO_4_, NH_4_Cl, and KNO_3_ treatments all showed significant decreases in *R*_m_ and *R*_n_ (P<0.05). The most obvious reduction effect occurred in the warm and rainy July. High level of (NH_4_)_2_SO_4_ addition had a stronger inhibition on *R*_m_ and *R*_n_ than NH_4_Cl and KNO_3_ addition. The slight promotion effect of (NH_4_)_2_SO_4_ in the early stages and inhibitory effect in the later stages of 2009 were also consistent with some previous results [12, 34, 35]. Zhang et al. [36] have reported that 3-year N addition (0–640 kg N ha^-1^ yr^-1^) caused gradual or step increases in soil NNM and nitrification in the early growing season. In a typical steppe dominated by a *Leymus chinensis* community, however, N addition also showed dose effects. Zhang et al.[37] found that low N addition (17.5 kg N ha^-1^ yr^-1^) stimulated N mineralization but high N addition (280 kg N ha^-1^ yr^-1^) inhibited N mineralization. Unfortunately, we did not measure soil *R*_m_ in the non-growing season of 2008 because of very low temperature and the deep snow, and only observed the slightly increased *R*_m_ in May 2009. N addition raised initial *R*_m_, because combination of the increased N with organic matter would decrease soil C/N ratio, accelerate the decomposition of SOM, and release nutrients [34]. Under the conditions of long-term fertilization, the total soil N mineralization would increase while the *R*_m_ would decline gradually [35,38]. Gundersen [39] also found that N input could elevate the *R*_m_ only in N-limited ecosystem. On the other hand, the inhibition might be that N input changes the chemical properties of SOM, and slows extracellular enzyme activity in the decomposition process [40,41]. The inhibited generation of humus degrades enzyme for the soil with elevated available N [12]. Liu et al. [42] found no effect of N fertilization on soil microbial indices in the first growing season in temperate steppe in northern China, which was not consistent with the results of N enrichment ecosystem [43]. Therefore, the responses of soil microbial activities to N forms should be further investigated in the N-limited steppe.

We found that KNO_3_ treatments decreased *R*_m_ and *R*_n_ in the later stages of 2009 (P<0.05). On the one hand, the addition of NO_3_^-^ directly inhibited nitrification. On the other hand, such decrease maybe correlated with the fact that NO_3_^-^ was added as KNO_3_ supplying with K^+^ ions. This K^+^ replaced H^+^ in soil exchange sites, leading to increase in the concentration of H^+^ in soil solution [44]. Recent research also showed that in N-limited ecosystems, NO_3_^-^ addition could cause slow decrease in soil pH [30]. N mineralization, especially nitrification, decreases linearly with the soil pH value [24,36]. Therefore, the effects of NH_4_^+^-N on the increment of soil N mineralization in the temperate semi-arid grassland is greater than that of NO_3_^—^N.

The reduction effects of N addition on *R*_m_ and *R*_n_ was most obvious in the warm and rainy July of 2009. The potential reason maybe that the optimal hydrothermal conditions favor soil denitrification, therefore decreased the *R*_m_ and *R*_n_.

In the two growing seasons, in either the LN or HN treatments, (NH_4_)_2_SO_4_ and KNO_3_ treatments did not significantly increase soil *R*_a_ in most of the period. However, the NH_4_Cl treatments significantly increased soil *R*_a_, except for some negative values (13 July-14 August 2008, 15 May-15 June, and 15 July-12 August 2009). Those negative values could be explained by N immobilization, or by gaseous losses at nitrification for higher moisture content due to the absence of plants in the field incubation experiment [21,45,46]. The negative *R*_a_ also indicates that the available NH_4_^+^-N could have been oxidized rapidly into NO_3_^—^N under the nitrification of autotrophic bacteria, or fixed by microorganism [3,47]. The promotion effects of NH_4_Cl on *R*_a_ maybe explained by the inhibition of Cl^-^ on the activity of soil microbial nitrification [28]. With the increase of NH_4_Cl, the promotion effect of HNH_4_Cl on *R*_a_ in 2008 was gradually disappeared in the later stages of 2009.

### The correlations between soil parameters and *R*_m_

While soil temperature and moisture are the main controlling factors of N mineralization, our results showed that *R*_m_ is positively correlated with SWC (P<0.05) but not significantly correlated with soil temperature (P>0.05). An explanation of these results is that our study area is located in a semi-arid climatic zone where SWC is relatively low. The mean air temperature of the area is higher than 10°C in most of the growing season. Some previous studies have shown that SWC limits NNM when SWC is less than 15%, and *R*_m_ rises with soil temperature in the temperature range from 5–35°C [48,49]. In our experiments, the SWC was less than 15% in most of the incubation period while daily mean air temperature was between 5–23°C (Fig 1), In such conditions, NNM was likely subjected to SWC.

There was a positive significant correlation between *R*_m_ and soil TN in our experiment results, this relationship was well documented and highlight the great impacts of TN on NNM [50]. Also, there was a highly significant relationship between soil *R*_m_ and T_a_ in different treatments of our experiments (Table 3, P<0.05). Some previous studies have also indicated that T_a_ could explain NNM by more than 83% [51]. The *R*_m_ was negatively but insignificantly correlated with C/N of SOM (P>0.05). N input did not change the soil C/N in short period in our experiments. A reason behind this result could be the fertilization time and N levels in soils. Vourlitis and Zorba [13] indicated that there was a significant negatively correlation between *R*_m_ and C/N of SOM. When the C/N of SOM is greater than 30:1, it may not provide N for plant in the initial stage of mineralization. When the C/N of SOM was less than 15:1 at the beginning of its mineralization, effective N provided by SOM will exceed the amounts of microbial assimilation, which makes it possible for plants to absorb the N from SOM mineralization [52].There is a positive but not significant correlation between *R*_m_ and soil pH values in our experiment results. Prior studies have shown that soil pH controlls the nitrification in soil [25,36]. Because soil pH could influence the dissolved organic matter, and the substances rich in carbon and N groups for microbial life activities, and therefore influence the N mineralization [6,47]. The correlations between *R*_m_ and environmental factors changed little in response to N addition (Table 3).

## Conclusions

With designed field experiments, this study investigated the short-term effects of N addition on soil mineral N and NNM. The results demonstrated that continuous two-year N addition had significantly affected soil mineral N and NNM. (NH_4_)_2_SO_4_ treatments increased soil NO_3_^-^ concentrations, while NH_4_Cl and KNO_3_ treatments decreased them. Three N treatments all decreased soil NH_4_^+^ concentrations. As for the NNM, three N fertilizer additions significantly decreased soil *R*_*m*_ over the short term, and the contribution of N addition to soil *R*_*m*_ was higher from (NH_4_)_2_SO_4_, than from NH_4_Cl and KNO_3_. However, we can’t expect the changes of soil NNM under the increased N deposition in the future. The long-term observations of N deposition effects on soil N transformation and the complex biochemical inhibition or promotion mechanisms by which inorganic N affects soil NNM are necessary.
